# Organocatalysis

**DOI:** 10.3762/bjoc.8.156

**Published:** 2012-08-23

**Authors:** Benjamin List

**Affiliations:** 1 Max-Planck-Institut für Kohlenforschung, Kaiser-Wilhelm-Platz 1, D-45470 Mülheim an der Ruhr, Germany

**Keywords:** organocatalysis

During the past 12 years or so, hardly any other field has impacted the art and science of chemical synthesis more profoundly than organocatalysis. The growth, both in academic research and in industrial use, has been breathtaking. Judging by the development of its impact factor, the *Beilstein Journal of Organic Chemistry* is currently attempting to achieve the same impact within chemistry publishing. A Beilstein Thematic Series on Organocatalysis therefore appeared to be quite logical. A brilliant opportunity arose in the context of a priority program sponsored by the Deutsche Forschungsgemeinschaft (DFG), which has in the meanwhile supported the organocatalysis research of over thirty groups in Germany between 2004 and 2010. The series in hand serves as the final report of this highly productive and successful “DFG Organokatalyse Schwerpunktprogramm, SPP 1179”. In addition, it captures the latest research activities in organocatalysis from many of the leading groups from all over the world.

Organocatalysis describes catalysis with low-molecular-weight organic compounds, in which a metal is not part of the active principle. Organocatalysts donate or remove electrons or protons as their activation mode, hence defining four distinct subareas: Lewis base and Lewis acid catalysis on the one hand, and Brønsted base and Brønsted acid catalysis on the other hand. There are additional aspects that belong to the field and that are being actively researched, including supported catalysts, organocatalysis in unusual reaction media, and applications in polymerizations, in total synthesis and drug-discovery, as well as in drug manufacturing, among many others. Potential advantages of organocatalysis have been emphasized on various occasions and will not be repeated here as such statements are becoming more and more inaccurate generalizations in light of the diversification and growth of the field. Suffice to say here that organocatalysis has plentiful and complementary reactivity to offer. Indeed, certain things that cannot be done with enzymes or metals may well be feasible with organic catalysts.

The present Thematic Series serves to showcase the current state of the art of organocatalysis and covers many of the aspects mentioned above. The productivity and creativity of the researchers in the field as such, and of the authors publishing here specifically, is truly amazing. I am highly grateful to all of the many contributors.

Ben List

Mülheim, August 2012

